# ID 116 - Evolução da Atuação dos Nats em Recomendações da Conitec entre 2018 e 2024

**DOI:** 10.5327/2237-9622.2025.v34s1.116

**Published:** 2025-11-25

**Authors:** Bernardo Salustio Pires, João Bratke, Bruno Boccia, Maria Fernanda Mussolino Ribeiro

**Keywords:** Nats, Conitec, Rebrats, Avaliação de Tecnologias em Saúde, protocolos clínicos

## Abstract

**Introdução:**

Os Núcleos de Avaliação de Tecnologias em Saúde (Nats) foram criados em 2009 com o objetivo de expansão dos conhecimentos de Avaliação de Tecnologias em Saúde (ATS) no Brasil. Além de sua atuação na promoção da ATS dentro das instituições de que fazem parte, os Nats também realizam, por meio de parcerias, análises e estudos para a Comissão Nacional de Incorporação de Tecnologias no SUS (Conitec). Com o aumento no número de demandas por avaliações na Conitec, as análises realizadas por parcerias com o Nats tornaram-se parte usual do processo de ATS no SUS. Para atuação junto à Conitec é necessário aos núcleos um nível de qualificação técnica e experiência em ATS. Este estudo buscou avaliar a evolução dos Nats com capacidade técnica para parcerias com a Conitec, com o objetivo de oferecer transparência sobre quais instituições estão envolvidas ao processo de ATS.

**Método:**

Os órgãos responsáveis pelas análises realizadas foram baseados nos responsáveis descritos na contracapa dos relatórios técnicos disponíveis no site da Conitec. A identificação dos órgãos responsáveis pelas análises foi baseada na contracapa dos relatórios disponibilizados pela Conitec, sendo considerado o primeiro órgão descrito como o principal contribuinte. Foram captados dados dos relatórios entre janeiro de 2018 e julho de 2024. Nesse período, os responsáveis pelas análises foram consistentemente identificados nos relatórios publicados.

**Resultados:**

Entre 2018 e 2024 um total de 20 Nats foram responsáveis pelas análises conduzidas nas avaliações da Conitec, dos quais dez participaram de avaliações de protocolos e diretrizes, 17 de medicamentos e 12 de procedimentos e outros produtos de saúde. No período, as análises realizadas por Nats passaram de uma minoria para estarem presentes na maioria dos relatórios, com apenas 7% dos relatórios em 2018 em comparação a 86% dos em 2024 declarando um Nats como principal elaborador das análises técnicas. O número de Nats parceiros cresceu principalmente desde 2022, período no qual nove novos núcleos realizaram análises à Conitec. Cerca de 56% das análises realizadas por Nats, entretanto, foram atribuídas a apenas três instituições: Uats/Haoc (Hospital Alemão Oswaldo Cruz), Nats/INC (Instituto Nacional de Cardiologia) e Nats/HMV (Hospital Moinhos de Vento), com 79, 24 e 16 análises, respectivamente. Similarmente, a Uats/Haoc é, isoladamente, responsável por 41% das análises de protocolos e diretrizes realizadas por Nats, com nove análises.

**Conclusão:**

Os Nats tornaram-se, desde 2018, parte essencial das avaliações da Conitec, tanto de protocolos quanto de tecnologias em saúde, com grande expansão no número de núcleos e quantidade de análises em que estes são envolvidos. Há ainda, entretanto, grande concentração de análises em alguns poucos núcleos, com oportunidade de expansão da atuação de outros.

